# A hypofractionated radiation regimen avoids the lymphopenia associated with neoadjuvant chemoradiation therapy of borderline resectable and locally advanced pancreatic adenocarcinoma

**DOI:** 10.1186/s40425-016-0149-6

**Published:** 2016-08-16

**Authors:** Todd Crocenzi, Benjamin Cottam, Pippa Newell, Ronald F. Wolf, Paul D. Hansen, Chet Hammill, Matthew C. Solhjem, Yue-Yun To, Amy Greathouse, Garth Tormoen, Zeljka Jutric, Kristina Young, Keith S. Bahjat, Michael J. Gough, Marka R. Crittenden

**Affiliations:** 1 Earle A. Chiles Research Institute, Robert W. Franz Cancer Center, Providence Portland Medical Center, 4805 NE Glisan St, Portland, OR 97213 USA; 2 The Oregon Clinic, Portland, OR 97213 USA; 3 Providence Hepatobiliary and Pancreatic Cancer Program, Providence Portland Medical Center, 4805 NE Glisan St, Portland, OR 97213 USA; 4 Oregon Health and Sciences University, Sam Jackson Parkway, Portland, OR USA

**Keywords:** Radiation, Fractionation, Chemotherapy, Gemcitabine, Lymphocytes, Lymphodepletion, Homeostatic repopulation, IL-7, IL-15, Immunotherapy

## Abstract

**Background:**

Preclinical studies have shown synergy between radiation therapy and immunotherapy. However, in almost all preclinical models, radiation is delivered in single doses or short courses of high doses (hypofractionated radiation). By contrast in most clinical settings, radiation is delivered as standard small daily fractions of 1.8-2 Gy to achieve total doses of 50–54 Gy (fractionated radiation). We do not yet know the optimal dose and scheduling of radiation for combination with chemotherapy and immunotherapy.

**Methods:**

To address this, we analyzed the effect of neoadjuvant standard fractionated and hypofractionated chemoradiation on immune cells in patients with locally advanced and borderline resectable pancreatic adenocarcinoma.

**Results:**

We found that standard fractionated chemoradiation resulted in a significant and extended loss of lymphocytes that was not explained by a lack of homeostatic cytokines or response to cytokines. By contrast, treatment with hypofractionated radiation therapy avoided the loss of lymphocytes associated with conventional fractionation.

**Conclusion:**

Hypofractionated neoadjuvant chemoradiation is associated with reduced systemic loss of T cells.

**Trial registration:**

ClinicalTrials.gov NCT01342224, April 21, 2011; NCT01903083, July 2, 2013.

**Electronic supplementary material:**

The online version of this article (doi:10.1186/s40425-016-0149-6) contains supplementary material, which is available to authorized users.

## Background

Surgical resection offers the only chance of cure for non-metastatic exocrine pancreatic cancer. However, only 15 to 20 % of patients have potentially resectable disease at diagnosis; approximately 40 % have distant metastases, and another 30 to 40 % have locally advanced unresectable tumors [1]. Typically, patients with locally advanced unresectable pancreatic cancer have tumor invasion into adjacent critical structures. The optimal management of these patients is controversial, and there is no standard approach [2]. Therapeutic options include radiation therapy alone, chemoradiotherapy, and chemotherapy alone. For those patients with locally advanced disease that precludes resection, or those patients with borderline resectable disease [3], neoadjuvant treatment represents an opportunity to reduce the size of the tumor sufficiently to allow an attempt at curative resection. Novel therapeutic regimens incorporating multiple chemotherapies have increased response rates amongst patients. In a direct comparison to Gemcitabine, FOLFIRINOX significantly increased overall survival from 6.8 months to 11.1 months in metastatic pancreatic cancer patients, but like gemcitabine does not result in durable cures [4]. This FOLFIRINOX regimen also results in increased hematological complications compared to gemcitabine [4]. The effects on T cells has not been reported, but based on the component chemotherapies this would be expected to be significant. Similarly, the addition of Abraxane to Gemcitabine for the treatment of metastatic pancreatic cancer significantly increased overall survival from 6.7 months to 8.5 months, though increased the rate of grade three or higher leukopenia and did not increase the number of durable cures [5]. Novel therapies are urgently needed for patients with pancreatic cancer, and we should consider how existing therapies can combine with immunotherapies that can target residual cancer cells to prevent local and distant recurrence. Cytotoxic therapies that result in the death of cancer cells also present an opportunity to use this treatment as an in situ cancer vaccine, to help in subsequent control of residual local and distant disease. In patients with other cancer types, immune responses arising from cytotoxic therapies combined with immunotherapies have been able to control extensive metastatic disease [6, 7], so using the patient’s own tumor to drive systemic immunity has a potential role in all patients [8, 9]. For these reasons it is critical to understand the effect of treatment on the immune system of patients with pancreatic adenocarcinoma.

It has long been known that traditional fractionated radiation can hinder immune responses [10] in part due to death of lymphocytes in the radiation field leading to systemic lymphodepletion [11]. Similarly, many chemotherapies display significant toxicity to lymphocyte populations *in vivo*. For this reason, there is broad concern that daily radiation treatments over the weeks of conventional therapy may be incompatible with generating immunity to tumors. Fractionation is a critical component of radiation therapy as it takes advantage of intrinsic radiobiological differences between cancer cells and normal cells, which is necessary when extensive areas of normal tissue are incorporated in the treatment field. More recently, through a combination of advanced imaging and advanced dose delivery, it has become possible to deliver high radiation doses to tumor while minimizing the volume of normal tissue at this high dose [12]. The relative benefit of these techniques remains a matter of discussion from the perspective of radiobiology, but from the perspective of immunology there are reasons to believe that causing acute rather than chronic antigen release would be preferred [13, 14], and chronic lymphocyte death in the tumor should be avoided. Fractionated chemoradiation therapy has been shown to result in lymphocyte loss in patients receiving treatment for pancreatic cancer [15], and other tumor types [16], and low lymphocyte counts following chemoradiation has been linked to poor treatment outcome [15]. Traditionally fractionated radiation in pancreatic cancer provides 1.8Gy per fraction for 28 fractions, resulting in a total dose of 50.4Gy, which is biologically equivalent to a hypofractionated course of 10Gy per fraction for 3 fractions [17]. This alternative regimen of hypofractionated radiation therapy has been shown to be similarly effective compared to fractionated radiation and safe in combination with chemotherapy for patients with pancreatic adenocarcinoma [18]. We hypothesized that hypofractionation would minimize the lymphocyte loss associated with chemoradiation and therefore represent a superior platform for future immunotherapy combinations.

To test this hypothesis, we examined two clinical studies at our institution. The first was a prospective study in 10 patients who received neoadjuvant chemoradiotherapy with gemcitabine and standard fractionated radiation therapy for locally advanced and borderline resectable pancreatic cancer. The second was a prospective study in 10 patients who also received combined modality therapy for locally advanced and borderline resectable pancreatic cancer but in the second study radiation therapy was delivered in a hypofractionated regimen. Using samples from these patients we were able to demonstrate lymphocyte loss in the peripheral blood associated with fractionated chemoradiation. We define the lymphocyte subpopulations affected and compare circulating levels of homeostatic cytokines in serum over the course of treatment. In addition we demonstrate intact cytokine signaling in circulating lymphocytes, suggesting that they are still responsive to homeostatic cytokines. Finally, for the first time we demonstrate significant lymphocyte preservation when radiation was administered in a hypofractionated regimen. These data provide compelling evidence that hypofractionated radiation may represent a superior partner for immunotherapy combinations, and demonstrates a feasible platform on which to build immunotherapy for treatment of patients with pancreatic cancer.

## Methods

### Patients

Patient blood samples were obtained from two sequential prospective clinical studies of neoadjuvant chemoradiotherapy in patients with locally advanced or borderline resectable pancreatic cancer. Both studies were approved by the institutional review board at Providence Portland Medical Center, Portland OR with study ID numbers PHS 10-141B and PHS 13-026A. The clinical trial registration numbers are NCT01342224 and NCT01903083. All patients provided informed consent for treatment and participation in these studies, including analysis of serum and blood parameters over the course of the study. In both studies, patients were treated with one cycle of gemcitabine pre-RT and received additional cycles of gemcitabine post-RT or post-operatively in those patients who were eligible for surgery.

For the first study, termed ICRT, patients were treated with intensity-modulated RT or 3D conformal technique to a total dose of 50.4 Gy in 28 fractions over 5.5 weeks. Gemcitabine was given concurrent with radiation at a dose of twice-weekly gemcitabine at 50 mg/m2. For the second study, termed CRIT, patients were treated with intensity-modulated RT to a total dose of 30 Gy in 3 fractions over one week. In both studies, gross tumor disease was treated and elective nodal irradiation was minimized. A treatment schematic of the two trials is provided in Additional file 1: Figure S1.

### Antibodies and reagents

For immunofluorescence staining of whole blood and PBMC, antibodies included CD45-ECD (Beckman Coulter, Indianapolis IN), CD3-e780, CD4-e450, CD8-AF700, CD15-FITC (eBioscience, San Diego, CA), CD45-FITC, CD4-PerCP-Cy5-5, CD14-PECy7, HLA-DR-APC, HLA-DR-BV421, CD8-APC-H7, CCR7-BV605, CD25-BV605, CD45RA-PECy7, CD45RO-APC-H7, CCR4-PECy7, CD38-APC, CD127-BV650 (BD Bioscience, San Jose, CA), CD14-AF700, and CD3-AF700 (Biolegend, San Diego, CA). For phosphoSTAT staining, antibodies included CD3-BV785 (Biolegend), CD4-BV605, CD8-BV510, CD14-PECy7, CD19-BV421, CD16-BV650, pSTAT3-647, pSTAT5-PE (BD Biosciences), and pSTAT1-488 (Cell Signaling Technologies, Danvers, MA). Cell types were identified according to the criteria of the Human Immunology Project [19], and a full list of antibodies used to identify individual cell types including those not reported here can be found in Additional file 2: Table S1. Hematological toxicity was graded according to NCI CTCAE v4.0 guidelines and is reported in Additional files 3 and 4: Tables S2 and S3.

### Flow cytometry

For analysis of cell numbers in blood, whole blood was collected in lavender top K_3_EDTA (ethylenediaminetetraacetic acid) collection tubes and stained directly with a pre-made cocktail of fluorescent antibodies in a tube with a known number of fluorescent beads (TruCOUNT Tube, BD Biosciences) within 4 h of collection. Following staining, red blood cells were lysed using FACS Lysing solution (BD Biosciences) and analyzed on a BD LSR II flow cytometer within 24 h of staining. The absolute number of positive cells (cells/μl) was calculated by comparing cellular events to bead events. Peripheral blood mononuclear cells (PBMC) were prepared from simultaneous blood samples and were cryopreserved within 2 h of collection for analyses of sub-phenotypes. Thawed PBMC were stained directly with a pre-made cocktail of fluorescent antibodies alongside quality control (QC) PBMC standards to ensure consistency of staining. QC values outside accepted parameters were discarded and all samples were restained.

### Cytokine bead assay

Serum was isolated from peripheral blood and cryopreserved. Cytokine levels in the serum were detected using a premixed human multiplex cytokine assay (Life Technologies, Grand Island, NY) or a custom Luminex performance human high sensitivity cytokine assay (R&D Systems, Minneapolis, MN) and read on a Luminex 100 array reader. Cytokine concentrations for replicates of each tumor sample were calculated according to a standard curve.

### Functional response to cytokines

PBMC were thawed, rested and exposed to appropriate cytokines for 15 min at 37 °C. Cytokine concentrations were 10^4^U/ml IFNα, 50 ng/ml IFNγ, 50 ng/ml IL-6, 50 ng/ml IL-7, 50 ng/ml IL-10, 50 ng/ml IL-21, and 50 ng/ml IL-2. Cells were then treated with paraformaldehyde for 10 min at room temperature, washed and permeabilized with cold methanol and stored at −80 °C before further analysis. To ensure comparable staining of all samples, each treatment group was stained with an individual combination of dyes to generate a unique barcode for identification on analysis [20, 21]. Thawed cells were left unstained, incubated with 0.2 μg/ml Pacific Orange (Life Technologies) or 0.03 μm/ml Pacific Orange, along with no further stain, 0.31 μg/μl Alexa 700 (Life Technologies) or 0.046 μg/ml Alexa 700. This creates 9 potential dye combinations such that each stimulation group had a unique combination of stains. Treatment groups were then washed and combined for surface staining with antibodies to distinguish the major cell types and intracellularly stained with directly conjugated antibodies for phospho-STATs. Samples were analyzed on a BD LSR II flow cytometer and each cell type was deconvoluted into treatment groups using BD FACSDiva software.

### Statistics

Data were analyzed and graphed using Prism (GraphPad Software, La Jolla, CA). Individual data sets were compared using Student’s *T*-test and analysis across multiple groups was performed using ANOVA with individual groups assessed using Tukey’s comparison.

## Results

### Patient demographics and radiotherapy compliance

20 patients with locally advanced or borderline resectable pancreatic cancer were initially enrolled in two sequential neoadjuvant studies at our institution. All patients were presented at a hepatobiliary-specific multi-disciplinary tumor board. Participation included radiologists, medical-, radiation-, and surgical-oncologists with a focus in pancreatic cancer. Staging was performed using a pancreatic protocol, multi-phasic CT scan and either a non-contrast chest CT or PET scan. Final determination of resectability was made by fellowship trained pancreatic surgeons based on NCCN guidelines [22]. Table 1 presents basic demographic and clinical information about the patients in each cohort. Patients enrolled into the ICRT (fractionated) study received 50.4 Gy of radiation in 28 daily fractions and patients enrolled into the CRIT (hypofractionated) study received 30 Gy of radiation in 3 fractions delivered over one week. In summary for the ICRT study median age was 61 y (range, 41–74), 6 patients were male and 4 were female, and 7 patients had locally advanced and 3 patients had borderline resectable disease. In the CRIT study median age was 64.5 y (range, 53–82), 5 patients were male and 5 were female, 2 patients had locally advanced and 8 patients had borderline resectable disease. 9 patients in the ICRT study completed the upfront gemcitabine and concurrent chemoradiation while one patient progressed prior to initiating chemoradiotherapy. 9 patients in the CRIT study completed the upfront gemcitabine and hypofractionated radiation while one patient progressed prior to initiating radiation. Patients who did not receive chemoradiation or hypofractionated radiation were not assessed for the effects of chemoradiation or hypofractionated radiation on immune cells. Of the 9 patients that initiated the concurrent chemoradiation on ICRT, all received the plan dose of 50.4Gy with a median elapsed days of 38.3 days, there were no treatment breaks for toxicity or machine malfunction. Of the 9 patients the initiated hypofractionated radiation, all received the plan dose of 30 Gy with a median elapsed days of 3.89 days, there were no treatment breaks for toxicity or machine malfunction.

**Table 1:** Patient characteristics

Study	ICRT (fractionated)		CRIT (Hypofractionated)	
Age	Median	Range	Median	Range
	61	41-74	64.5	53-82
Sex	Male	Female	Male	Female
	6 (60.0 %)	4 (40.0 %)	5 (50.0 %)	5 (50.0 %)
Diagnosis	Locally advanced	Borderline resectable	Locally advanced	Borderline resectable
	7 (70.0 %)	3 (30.0 %)	2 (20.0 %)	8 (80.0 %)
Race	Caucasian	All other	Caucasian	All other
	10 (100 %)	0 (0 %)	9 (90 %)	1 (10 %)
Stage	IIA-IIB	III-IV	IIA-IIB	III-IV
	4	5	7	2
Surgery	Yes	No	Yes	No
	4	5	6	3

### Fractionated radiation reduces lymphocyte counts including both the CD4 and CD8 compartment

**Fig. 1:**
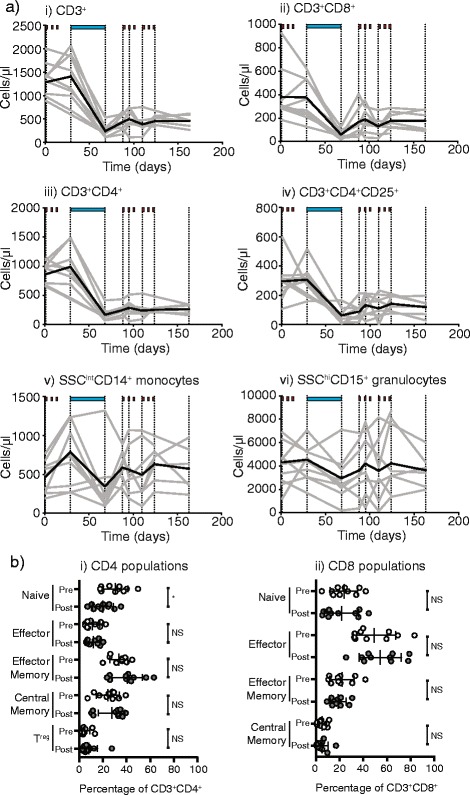
Effect of conventional neoadjuvant chemoradiation on immune cells in patient blood. **a** Absolute numbers of i) CD3^+^, ii) CD8^+^, iii) CD4^+^ and iv) CD4^+^CD25^+^ T cells as well as v) SSC^int^CD14^+^ monocytes and vi) SSC^hi^CD15^+^ granulocytes by flow cytometry of fresh peripheral blood over the course of the study. Individual patients are gray, the mean is black. Dotted lines show sampling times and shows periods of neoadjuvant and adjuvant chemotherapy (*red rectangles*), neoadjuvant chemoradiation (*blue bar*) according to the trial schema provided in Additional file 1: Figure S1. **b** Analysis of the proportions of i) CD4 and ii) CD8 subpopulations in PBMC collected pretreatment (*open circles*) and post-treatment (*closed circles*). Key: NS – not significant. * *p* < 0.05

Quantitative analysis of peripheral blood immune cells through the ICRT (fractionated chemoradiation) study treatment demonstrated that the number of CD3^+^ T cells in all patients were dramatically reduced in frequency (Fig. 1a). Both CD3^+^CD8^+^ and CD3^+^CD4^+^ T cells as well as the CD3^+^CD4^+^CD25^+^ population that includes T regulatory cells were dramatically reduced in number and remained so for the duration of the study (Fig. 1a). This decline was not evident through the initial cycles of chemotherapy, but was observed during the fractionated chemoradiation. Myeloid populations remained broadly unchanged at the end of treatment. To determine whether specific subtypes of T cells were affected in this decline, naïve, effector and memory cells were distinguished using the markers CD45RO, CD27 and CD28 as were T regulatory cells using the markers CD25 and CD127. There were no changes in the proportion of CD8 T cell subtypes, indicating that all were decreased equivalently (Fig. 1b). There were significant decreases in the proportion of naïve CD4 T cells that was not compensated by a significant change in any other individual T cell populations (Fig. 1b), but if effector memory and central memory populations are combined these memory cells significantly increase in proportion (*p* < 0.05). Thus, amongst the general T cell decline there was a proportional switch resulting in fewer naïve CD4 T cells and more memory CD4 T cells, even though both declined in number. To determine whether these lymphocytes ever recovered, patients were followed by complete blood count on return visits. Blood counts show the loss of lymphocytes and recovery periods extending up to 2 years before normalization (Table 2). These data demonstrate that conventionally fractionated radiation in combination with chemotherapy resulted in a dramatic and prolonged decrease in T cells in the peripheral blood of patients.

**Table 2:** Normalization of absolute lymphocyte counts by CBC

STUDY	ICRT (Fract)	CRIT (Hypo)
Percent of patients with normal ALC for 2 consecutive measurements post RT	40 % (4/10)	70 % (7/10)
Mean time to normal ALC for patients that normalize	272 days (108–523 days)	50 days (all patients)

### Gamma common chain homeostatic cytokine levels remain stable or increased through treatment

**Fig. 2:**
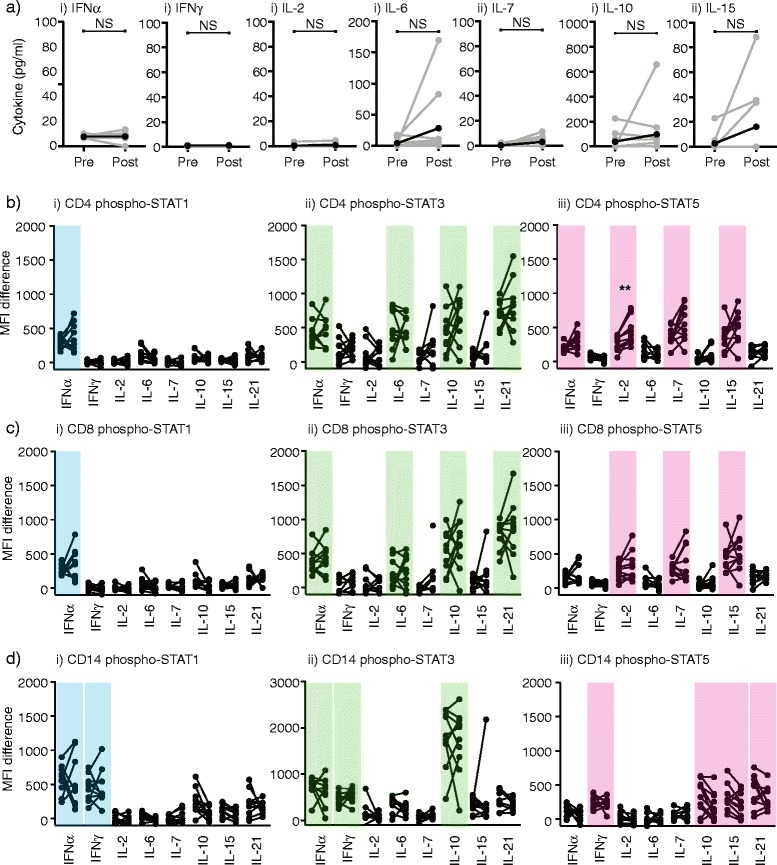
Role of homeostatic repopulation through cytokines and cytokine responses. **a** Patient serum collected prior to treatment (pre) and at the end of treatment (post) were tested for cytokine levels by multiplex assay. Individual patients are gray, the mean is black. **b-d** PBMC collected prior to treatment and at the end of treatment were treated with a range of cytokines for and analyzed for i) pSTAT1, ii) pSTAT3 or iii) pSTAT5 expression by intracellular flow cytometry. Surface staining of these mixed populations identified pSTAT activation in (**b**) CD4^+^ T cells, (**c**) CD8^+^ T cells or (**d**) CD14^+^ monocytes. Individual patient’s pre and post treatment values are connected. Graphs show change in pSTAT MFI over control (vehicle alone) stimulated cells. Colors highlight STATs that respond to a particular stimulation in each cell type. Key: NS – not significant. * *p* < 0.05, ** *p* < 0.01

Some immunotherapy strategies intentionally deplete lymphocytes by chemotherapy and total body radiation therapy in order to take advantage of the homeostatic repopulation of lymphocytes that follows [23–25]. In this setting, a relative abundance of homeostatic cytokines [23] causes costimulation-independent [26] expansion of lymphocytes to recover the lymphoid pool to pre-treatment numbers [27]. To understand the failure of homeostatic repopulation of T cells in our patients undergoing chemoradiation, we measured key cytokines in the serum of patients before treatment and when treatment was completed to determine whether there was a lack of production of homeostatic cytokines. We found that there was no lack of the critical homeostatic cytokines IL-7, IL-15 and IL-2, and in fact some patients displayed elevated levels of these cytokines post-treatment but sustained low T cell counts (Fig. 2). This means that on a per cell basis, the homeostatic cytokines are more available to T cells during lymphopenia, which has been hypothesized to direct their recovery [23].

### Functionally intact cytokine signaling in T cells following fractionated radiation

Alternatively, there could be some functional defect in the residual T cells rendering them unable to respond to cytokine-driven homeostatic expansion. To determine whether there was a functional deficit in the T cells, we developed a multiplex assay for cytokine signaling in patient PBMC using phospho-STAT activation (based on the techniques reviewed in [20, 21]). PBMC were separated into pools and ‘barcoded’ with unique combinations of viable dyes and each pool was stimulated with a specific cytokine. Following stimulation cells were washed and combined for surface staining and intracellular p-STAT staining to ensure highly reproducible staining between the treatment groups. Following flow cytometry, samples could then be deconvoluted by ‘barcode’ and surface phenotype to analyze responses to each stimuli in each cell type (Fig. 2). Each cytokine resulted in a particular pattern of STAT activation depending on the cell type, and responses were consistent with receptor expression. For example, only monocytes responded to IFNγ with STAT1 activation, all cells responded to IL-10 with STAT3 activation but not STAT1 or STAT5, while CD8 and CD4 T cells but not monocytes treated with IL-7 responded exclusively with STAT-5 (Fig. 2). By comparing patient PBMC before and after treatment, we saw no decrease in the functional response to any cytokine, in fact CD4 T cells showed significantly higher STAT5 activation with IL-2 stimulation following therapy, and the STAT5 response to IL-7 increased in individual patients but did not achieve significance as a group (Fig. 2). These data demonstrate that homeostatic cytokines are not decreased following therapy and there is no functional impairment in cytokine signaling in T cells. These data suggest that the principles of rapid homoeostatic repopulation of lymphocytes following transient lymphopenia do not apply to the more prolonged lymphopenia that occurs following fractionated radiation combined with chemotherapy.

### Hypofractionated radiation is not associated with significant long term lymphocyte loss

**Fig. 3:**
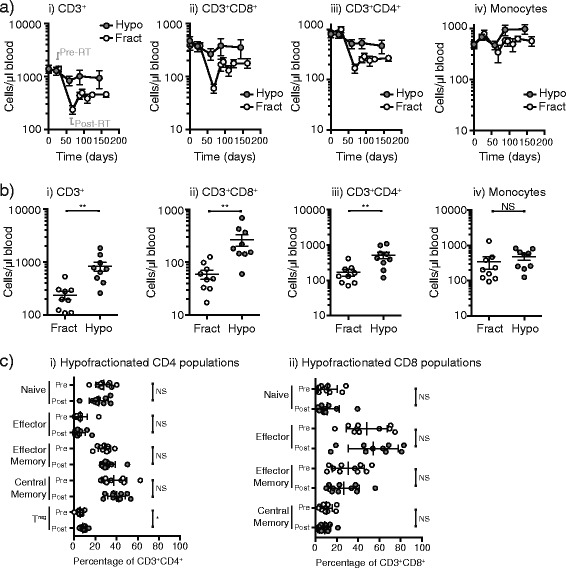
Effect of hypofraction on immune cells in patient blood. **a** Absolute numbers of i) CD3^+^, ii) CD8^+^, iii) CD4^+^ and iv) SSC^int^CD14^+^ monocytes by flow cytometry of fresh peripheral blood in patients on a conventionally fractionated regimen (Fract, open symbols) versus a hypofractionated regimen (Hypo, closed symbols). The samples immediately pre-RT and immediately post-RT are highlighted on the first graph. **b** comparison of absolute numbers of i) CD3^+^, ii) CD8^+^, iii) CD4^+^ and iv) SSC^int^CD14^+^ monocytes by flow cytometry of fresh peripheral blood immediately following completion of radiation therapy in patients on a conventionally fractionated regimen (Fract, open symbols) versus a hypofractionated regimen (Hypo, closed symbols). **c** Analysis of the proportions of i) CD4 and ii) CD8 subpopulations in PBMC collected pretreatment (*open circles*) and post-treatment with a hypofractionated regimen (*closed circles*)

Quantitative analysis of peripheral blood immune cells through CRIT (hypofractionated chemoradiation) study treatment demonstrated that myeloid populations again remained broadly unchanged through treatment, and CD3^+^ T cell populations declined but this did not achieve statistical significance (Fig. 3a). Comparison of T cell subpopulations in patients in the conventionally fractionated versus the hypofractionated regimen showed that CD3^+^CD8^+^ and CD3^+^CD4^+^ T cells were substantially higher in number following treatment in patients receiving hypofractionated chemoradiation as compared to patients treated with fractionated chemoradiation (Fig. 3b). Similar to patients with conventionally fractionated radiation, we examined the T cell subpopulations in patients treated with hypofractionated radiation and found few significant changes in the proportion of CD8 T cell subtypes or CD4 T cell subtypes. The change in the proportion of naïve (CD27^+^CD28^+^) CD4 T cells seen with conventionally fractionated chemoradiation was not seen with hypofractionated radiation and there was no compensatory increase in memory cells, but in the hypofractionated patients we did see a slight but statistically significant increase in the proportion of T regulatory cells (Fig. 3c). By examining absolute lymphocyte counts from CBC measurements in patients following treatment extending beyond the study period, we can see that the majority of patients receiving conventionally fractionated radiation commonly fail to normalize their lymphocyte count, and where they do it can take up to 2 years to normalize (Table 2). By contrast, the majority of patients receiving hypofractionated radiation normalize their ALC and they do so within the treatment period. These data demonstrate that hypofractionated chemoradiation is significantly less lymphodepleting than conventionally fractionated radiation, and therefore may be a better partner for combination with immune therapies and may avoid the poor prognosis associated with low lymphocyte counts.

### Comparison of gamma common chain cytokines between hypofractionated and standard fractionated treatment

**Fig. 4:**
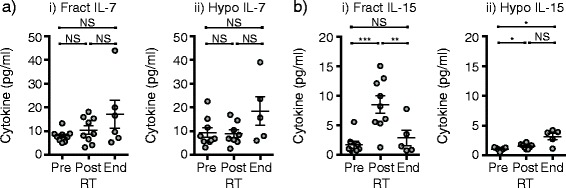
Effect of treatment regimen on early homeostatic cytokines. Patient serum collected prior to treatment (pre), immediately following completion of radiation therapy (post) and at the end of treatment (end) from patients on i) a conventionally fractionated regimen (Fract) versus ii) a hypofractionated regimen (Hypo) were tested for (**a**) IL-7 and (**b**) IL-15 cytokine levels by ultrasensitive multiplex assay. Key: NS – not significant. * *p* < 0.05, ** *p* < 0.01, *** *p* < 0.001

It is possible that hypofractionated radiation avoids lymphodepletion though upregulation of homeostatic cytokines beyond that seen in conventional fractionation. To assess this, we measured serum cytokines in the two groups of patients using an ultrasensitive assay, and included serum samples collected immediately following completion of the radiation treatment where T cells are at their nadir. We measured no significant change in the homeostatic cytokine IL-7 over time with either radiation treatment (Fig. 4ai-ii), but we did observe an increase in IL-15 in patients in both conventionally fractionated and hypofractionated patients (Fig. 4bi-ii), though it increased more in the conventionally fractionated rather than hypofractionated patients. Comparing the two groups, IL-15 levels in the serum at the post-RT lymphocyte nadir is the only statistically significant difference between conventionally fractionated and hypofractionated patients (*p* < 0.001). These data indicate that cytokine levels in patients receiving hypofractionated radiation are not sufficient to explain the difference in long-term lymphocyte numbers between the two treatment regimes. However, this data does suggest that homeostatic cytokine levels might explain some portion of the early recovery from lymphopenia in the conventionally fractionated patients, though the patients appear to settle at a new lymphocyte number set point that stops short of a full recovery to pre-treatment levels over time.

### Role of treatment volume in lymphopenia

**Fig. 5:**
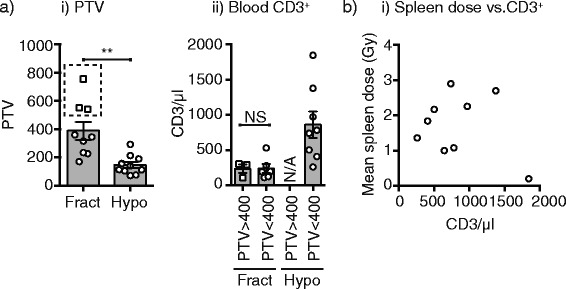
Link between planning target volume (PTV), spleen dose and T cell count. **a**) i) Graph showing the PTV for patients receiving a conventionally fractionated regimen (Fract) versus a hypofractionated regimen (Hypo). A subset of patients receiving a conventionally fractionated regimen exhibited a PTV greater than 400 (square symbols). ii) Post-chemoRT blood T cell counts are shown for patients with PTV greater than or less than 400 receiving a fractionated regimen (Fract) versus a hypofractionated regimen (Hypo). **b**) Relationship between mean spleen dose (Gy) and post-chemoRT blood T cell counts for patients receiving a hypofractionated regimen. Each symbol represents one patient. Key: NS – not significant. ** *p* < 0.01

The small sample size of these studies means that we cannot determine whether the degree of lymphopenia is caused by the treatment volume. While the PTV is significantly smaller in hypofractionated treatment plans, those patients in the fractionated group who have comparable treatment volume to those in the hypofractionated group still display lower lymphocytes (Fig. 5a). Analysis of a much larger sample set will be required to determine if the mechanism by which hypofractionation avoids lymphocyte loss is exclusively due to treatment volume. Within patients receiving hypofractionated radiation, there was no association between splenic dose and lymphocyte number (Fig. 5b).

## Discussion

These data demonstrate that there is a prolonged T cell lymphopenia associated with conventionally fractionated chemoradiation therapy for patients with pancreatic adenocarcinoma, which we propose makes this treatment regimen a poor partner for immunotherapies targeting T cells. We show for the first time in patients that the lymphopenia of chemoradiation can be avoided using a hypofractionated radiation regimen, suggesting this approach may be a superior partner for immunotherapy targeting T cells.

Traditionally fractionated radiation treatment of cancer has been known to can cause lymphocyte loss for approximately 40 years, and this issue remains despite dramatic advances in the targeting and delivery of radiation to patients. Importantly, lymphocyte loss has been observed in a range of tumor types and locations [10, 15, 16, 28–30], encompassing a range of delivery methods and chemotherapy partners. Broadly, the effect of therapy on lymphocytes has not been a major consideration in the design of treatment regimens. Recent data indicating that systemic lymphocyte and myeloid populations can be differentially affected by radiation therapy [31, 32], suggests that it may be possible to refine radiation delivery for immune consequences in addition to death of cancer cells. It may be possible to reduce lymphocyte loss through close control of PTV [30], but we also propose that this refinement should consider dose and fractionation. While others have described that lymphocyte declines are associated with poor prognosis in patients with pancreatic cancer, these assays were performed using complete blood counts and we do not know whether the decline involves T lymphocytes and their subpopulations [15]. However, in a study of patients with head and neck carcinoma, those undergoing treatment with cisplatin or carboplatin along with fractionated radiation were found to have fewer CD4 and CD8 T cells in the blood than those untreated or receiving surgical treatment [33]. T regulatory cells were not found to change in number following treatment, but did therefore increase as a proportion of CD4 or in relation to CD8 [33]. Though in the aforementioned study, T regulatory cell numbers did not influence tumor recurrence, these T regulatory cells were functional and they may have the potential to impact immunotherapies targeting T cells. Patients with high grade glioma undergoing treatment with temozolomide and fractionated radiation were found to have a lymphocyte decrease due only to loss of CD4 T cells, with the CD8 T cells remaining relatively stable [34]. As found in our study, at later time points in these patients loss of lymphocytes was not associated with a change in IL-7 or IL-15 cytokine levels in the peripheral blood. Similar to the head and neck carcinoma literature, T regulatory cells were found to increase as a proportion of CD4 but did not increase in number due to the overall decrease in CD4 T cells [34]. Since we also observe a proportional increase in T regulatory cells following hypofractionated radiation therapy, this may provide a mechanistic rationale for treatments that decrease T regulatory cells in patients in conjunction with hypofractionated radiation therapy. In mouse models, depletion of T regulatory cells synergizes with hypofractionated radiation therapy for improved tumor control [35]. In addition, therapy using anti-CTLA4, which depletes T regulatory cells in the tumor as a necessary mechanism of action [36, 37], synergizes with hypofractionated radiation therapy in mouse models [38, 39] and a number of clinical anecdotes have been reported where anti-CTLA4 plus hypofractionated palliative radiation have resulted in systemic tumor cure [7].

Standard treatment for locally advanced, borderline resectable pancreatic cancer and metastatic pancreatic cancer is rapidly evolving and the standard treatment is still being defined. Current approaches in the locally advanced, border line resectable setting include chemotherapy alone with either FOLFIRINOX or Gemcitabine and nab-paclitaxel (Gem/Nab) and in non-progressing patients consideration of RT followed by resection in borderline resectable patients [2] Increasingly, hypofractionated regimens of RT are being studied in the neoadjuvant RT setting for both biological effect as well as to shorten RT treatment time to transition patients to surgery faster [40, 41]. Hypofractionated dosing for neoadjuvant treatment of pancreatic cancer remains experimental and has not been standardized, but early phase clinical studies have demonstrated that it is well tolerated, with comparable efficacy to historical controls [18]. These hypofractionated techniques typically deliver RT in doses of 6-10Gy in 3–5 treatments and limit fields to areas of gross disease alone. In the metastatic setting, systemic FOLFIRINOX and or Gem/Nab chemotherapy is most frequently used. RT in the metastatic setting is used for palliation and with a range of doses and fractionations. The standard course of chemotherapy in both studies included cycles of gemcitabine before and after radiation therapy. These treatment cycles had no effect on lymphocyte numbers. Chemotherapy drugs have a variable effect on immune cells. These range from myelotoxic chemotherapies such as 5-FU to lymphotoxic chemotherapies such as cyclophosphamide. A number of investigators are testing the combination of immunotherapy with chemotherapy (reviewed in [42]) with variable success [43–45]. It is likely that just as there are optimal doses and selections of chemotherapy that succeed as partners for immunotherapy, there are likely optimal doses and fractionations of radiation that can similarly partner with immunotherapy. While gemcitabine is generally found to be non-lymphodepleting, the FOLFIRINOX cocktail incorporates agents known to cause lymphocyte loss. It is particularly encouraging that in mouse models, systemic Gem/Nab chemotherapy plus immunotherapy was able to initiate sufficient anti-tumor immune responses for tumor control with anti-CTLA4 plus anti-PD1 therapy [46]. Each variation of chemotherapy and radiation therapy has the potential to kill cancer cells and release antigen thus functioning as an *in situ* vaccine. The relative ability of the range of available approaches to synergize with immunotherapy in patients remains to be determined. Since most patients on the fractionated chemoradiation study died before lymphocyte normalization, the optimal window for immunotherapy in combination with fractionated chemoradiation likely needs to occur before, or very soon after initiation of combined therapy. Further studies will be needed to establish the precise timing of lymphocyte loss through fractionated chemoradiation to determine whether such a window exists. We recently demonstrated that anti-CTLA4 immunotherapy was optimal when delivered before radiation therapy [47], though, as with most preclinical work on radiation therapy and immunotherapy, this was tested using a hypofractionated regimen and chemotherapy was not present.

Using simulation, Yovino et al. [30] demonstrated a link between field size, the number of fractions and the dose rate in predicting blood exposure to radiation. Our hypofractionated treatment plans incorporated a significantly smaller PTV than the conventionally fractionated plans, thus the smaller PTV could explain reduced lymphopenia. However, where there were patients in each group with similar PTV, they exhibited prolonged lymphopenia following conventional fractionation but not following hypofractionation. In addition, these data are influenced by the requirement for a much steeper dose fall-off in hypofractionated treatments to minimize potential damage to nearby sensitive structures. These data hint that PTV alone is not sufficient to explain the data; however, a much larger sample size will be needed to make this conclusion. The mechanism by which hypofractionated radiation avoids the prolonged lymphodepletion in combination with chemotherapy remains to be determined. Amongst the potential explanations, as discussed, it could be due to the distinct treatment volume in the treatment groups, alternatively it could also be due to the fact that radiation treatment is completed in less than 1 week as compared to 5.5 weeks. Nevertheless, if lymphocyte numbers in the normal range are required for additional therapies, for example immunotherapy, this course of fractionated chemoradiation should be avoided. The exclusivity of the effect to the lymphocyte subpopulations suggests that this is not an effect of the hematopoietic stem cells in the bone marrow which would be expected to impact additional hematopeietic populations. In addition lymphocyte homeostasis in adults is driven by lymphocyte proliferation in secondary lymphoid organs.

Neoadjuvant and adjuvant therapies have different implications for tumor immunity. In the neoadjuvant setting, radiation is mostly targeted to gross tumor whereas in the adjuvant setting radiation is targeted to the resection site consisting mostly of healing normal tissue. In adjuvant treatment, tumor antigen release from the few residual cancer cells is likely to have less of an impact on initiating immune responses as in order to prime immunity through cytotoxic therapy, cancer cells must be present to provide antigens. Indeed, in mouse models combining immunotherapy in conjunction with surgical resection of tumors, we found that the opportunity to prime anti-tumor T cell responses was rapidly lost after the tumor was resected [48]. This implies an additional role for neoadjuvant treatment to generate immunity in addition to its usual role of killing cancer cells. Immunohistochemical analysis of pancreatic tumors has shown relatively poor infiltration of T cells, and poor prognosis where T cell numbers are low and macrophage numbers are high [49]. While it is logical to believe that a log lower number of T cells in the periphery will result in fewer T cells in the tumor, it remains to be determined whether this is the case in post-chemoradiation lymphopenia. Nevertheless, Campian et al. have demonstrated that systemic lymphopenia is associated with worse prognosis in patients with pancreatic adenocarcinoma [50]. Similarly, it remains to be determined whether these lymphopenic patients are less responsive to T cell targeted immunotherapy, though there is some data that pre-treatment T cell number is a predictor of outcome following anti-CTLA4 immunotherapy [51]. Due to the extreme resistance of pancreatic cancer to treatment, for the forseeable future the use of neoadjuvant therapy is unlikely to diminish the need for curative resection in pancreatic cancer. However, in addition to increasing the number of candidates eligible for resection, neoadjuvant therapy may be viewed as a an opportunity for combination immunotherapy to decrease local and distant recurrence by boosting immune recognition and targeting of residual cancer cells. In that setting, we propose that hypofractionated radiation regimens are most appropriate.

## Conclusions

Neoadjuvant chemoradiation with standard fractionation is associated with durable loss of lymphocytes in patients with pancreatic cancer. In the emerging era of clinical immunotherapies, this conventional approach may be an inferior partner with treatments that rely on T lymphocytes for their action. We demonstrate that radiation therapy administration with a hypofractioned regimen preserves lymphocytes and may therefore be a superior partner for combination with immunotherapies. This has implications regarding the design of future clinical studies combining immunotherapy with conventional therapies.

## Abbreviations

CD, cluster of differentiation; Gy, gray; IFN, interferen; IL, interleukin; PBMC, peripheral blood mononuclear cells

## Additional files

Additional file 1: Figure S1. Organization of clinical studies. Patients with locally advanced, unresectable or borderline resectable pancreatic adenocarcinoma with absence of distant metastatic disease were eligible for a) Conventional neoadjuvant chemoradiation scheme. b) Neoadjuvant chemoradiation with a hypofractionated scheme. In each study patients are assessed for potential resection following initial treatment and where eligible receive pancreaticoduodenectomy 4–8 weeks after the last dose of radiation therapy. Patients ineligible for resection proceed to futher treatment, those receiving resection receive further treatment 4–12 weeks following the operation. (PDF 233 kb) Additional file 2: Table S1. Phenotyping of peripheral blood immune cells. (DOCX 16 kb) Additional file 3: Table S2. NCI CTCAE v4.0 hematological toxicity - fractionated. (DOCX 20 kb) Additional file 4: Table S3. NCI CTCAE v4.0 hematological toxicity - hypofractionated. (DOCX 20 kb)
