# Supplementary figures and images for: A hypofractionated radiation regimen avoids the lymphopenia associated with neoadjuvant chemoradiation therapy of borderline resectable and locally advanced pancreatic adenocarcinoma
Source: J Immunother Cancer. 2016 Aug 16;4:45. doi: 10.1186/s40425-016-0149-6 (PMC4986363; doi:10.1186/s40425-016-0149-6)

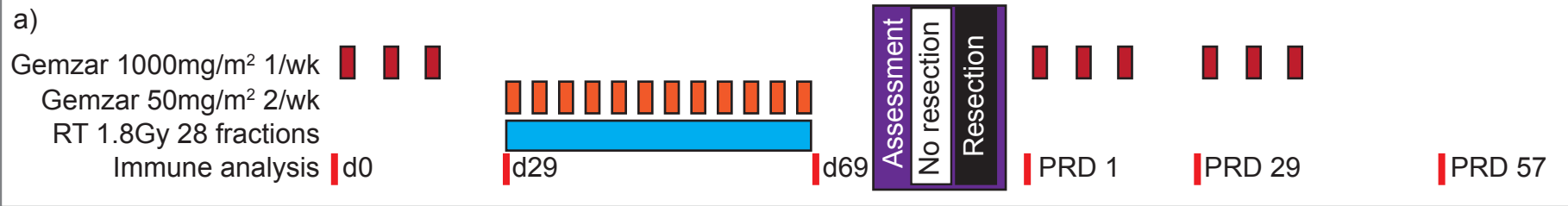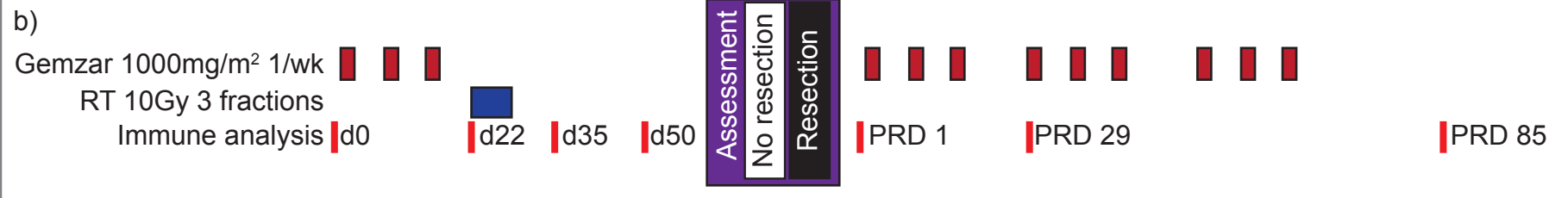

Supplement: Additional file 1: Figure S1. — Organization of clinical studies. Patients with locally advanced, unresectable or borderline resectable pancreatic adenocarcinoma with absence of distant metastatic disease were eligible for a) Conventional neoadjuvant chemoradiation scheme. b) Neoadjuvant chemoradiation with a hypofractionated scheme. In each study patients are assessed for potential resection following initial treatment and where eligible receive pancreaticoduodenectomy 4–8 weeks after the last dose of radiation therapy. Patients ineligible for resection proceed to futher treatment, those receiving resection receive further treatment 4–12 weeks following the operation. (PDF 233 kb) [file 40425_2016_149_MOESM1_ESM.pdf]
