# Supplementary material for: A hypofractionated radiation regimen avoids the lymphopenia associated with neoadjuvant chemoradiation therapy of borderline resectable and locally advanced pancreatic adenocarcinoma
Source: J Immunother Cancer. 2016 Aug 16;4:45. doi: 10.1186/s40425-016-0149-6 (PMC4986363; doi:10.1186/s40425-016-0149-6)
Supplement: Additional file 2: Table S1. — Phenotyping of peripheral blood immune cells. (DOCX 16 kb) [file 40425_2016_149_MOESM2_ESM.docx]

Additional file 1:  **Table S1: Phenotyping of peripheral blood immune cells**

T cells (CD3+)

CD4+ T cells (CD3+CD4+)

Activated CD4+ T cells (CD3+CD4+CD38+HLA-DR+)

Naïve CD4+ T cells (CD3+CD4+CCR7+CD45RA+)

Activated Naïve CD4+ T cells (CD3+CD4+CCR7+CD45RA+CD38+HLA-DR+)

Central Memory CD4+ T cells (CD3+CD4+CCR7+CD45RA-)

Activated Central Memory CD4+ T cells (CD3+CD4+CCR7+CD45RA-CD38+HLA-DR+)

Effector CD4+ T cells (CD3+CD4+CCR7-CD45RA+)

Activated Effector CD4+ T cells (CD3+CD4+CCR7-CD45RA+CD38+HLA-DR+)

Effector Memory CD4+ T cells (CD3+CD4+CCR7-CD45RA-)

Activated Effector Memory CD4+ T cells (CD3+CD4+CCR7-CD45RA-CD38+HLA-DR+)

CD8+ T cells (CD3+CD8+)

Activated CD8+ T cells (CD3+CD8+CD38+HLA-DR+)

Naïve CD8+ T cells (CD3+CD8+CCR7+CD45RA+)

Activated Naïve CD8+ T cells (CD3+CD8+CCR7+CD45RA+CD38+HLA-DR+)

Central memory CD8+ T cells (CD3+CD8+CCR7+CD45RA-)

Activated Central Memory CD8+ T cells (CD3+CD8+CCR7+CD45RA-CD38+HLA-DR+)

Effector CD8+ T cells (CD3+CD8+CCR7-CD45RA+)

Activated Effector CD8+ T cells (CD3+CD8+CCR7-CD45RA+CD38+HLA-DR+)

Effector Memory CD8+ T cells (CD3+CD8+CCR7-CD45RA-)

Activated Effector Memory CD8+ T cells (CD3+CD8+CCR7-CD45RA-CD38+HLA-DR+)

CD4-CD8- T cells (CD3+CD4-CD8-)

Activated CD4-CD8- T cells (CD3+CD4-CD8-CD38+HLA-DR+)

Naïve CD4-CD8- T cells (CD3+CD4-CD8-CCR7+CD45RA+)

Activated Naïve CD4-CD8- T cells (CD3+CD4-CD8-CCR7+CD45RA+CD38+HLA-DR+)

Central memory CD4-CD8- T cells (CD3+CD4-CD8-CCR7+CD45RA-)

Activated Central Memory CD4-CD8- T cells (CD3+CD4-CD8-CCR7+CD45RA-CD38+HLA-DR+)

Effector CD4-CD8- T cells (CD3+CD4-CD8-CCR7-CD45RA+)

Activated Effector CD4-CD8- T cells (CD3+CD4-CD8-CCR7-CD45RA+CD38+HLA-DR+)

Effector Memory CD4-CD8- T cells (CD3+CD4-CD8-CCR7-CD45RA-)

Activated Effector Memory CD4-CD8- T cells (CD3+CD4-CD8-CCR7-CD45RA-CD38+HLA-DR+)

T cells (CD3+)

CD4+ T cells (CD3+CD4+)

Activated CD4+ T cells (CD3+CD4+CD38+HLA-DR+)

CD8+ T cells (CD3+CD8+)

Activated CD8+ T cells (CD3+CD8+CD38+HLA-DR+)

Th1 cells (CD3+CD4+CXCR3+CCR6-)

Activated Th1 cells (CD3+CD4+CXCR3+CCR6-CD38+HLA-DR+)

Th2 cells (CD3+CD4+CXCR3-CCR6-)

Activated Th2 cells (CD3+CD4+CXCR3-CCR6-CD38+HLA-DR+)

Th17 cells (CD3+CD4+CXCR3-CCR6+)

Activated Th17 cells (CD3+CD4+CXCR3-CCR6+CD38+HLA-DR+)

Th17 cells (CD3+CD4+CXCR3+CCR6+)

Activated Th17 cells (CD3+CD4+CXCR3+CCR6+CD38+HLA-DR+)

T cells (CD3+)

CD4+ T cells (CD3+CD4+)

T Reg cells (CD3+CD4+CCR4+CD25+CD127low)

Naïve T Reg cells (CD3+CD4+CCR4+CD25+CD127lowCD45RO-)

Activated Naïve T Reg cells (CD3+CD4+CCR4+CD25+CD127lowCD45RO-HLA-DR+)

Memory T Reg cells (CD3+CD4+CCR4+CD25+CD127lowCD45RO+)

Activated Memory T Reg cells (CD3+CD4+CCR4+CD25+CD127lowCD45RO+HLA-DR+)

Monocytes (CD14+CD3-CD19-CD20-)

Non-classical CD16+ monocyte (CD14+CD16+CD3-CD19-CD20-)

Classical monocyte (CD14+CD16-CD3-CD19-CD20-)

Total DC (HLA-DR+CD14-CD3-CD19-CD20-)

Plasmacytoid DC (HLA-DR+CD123+CD14-CD3-CD19-CD20-)

Myeloid DC (HLA-DR+CD11c+CD14-CD3-CD19-CD20-)

CD16+ Myeloid DC (HLA-DR+CD11c+CD16+CD14-CD3-CD19-CD20-)

CD16- Myeloid DC (HLA-DR+CD11c+CD16-CD14-CD3-CD19-CD20-)

Total NK cells (CD16+ or CD56+ CD14-CD3-CD19-CD20-)

CD16+CD56- NK cells (CD16+CD56-CD14-CD3-CD19-CD20-)

CD16+CD56+ NK cells (CD16+/-CD56brightCD14-CD3-CD19-CD20-)

CD16-CD56bright NK cells (CD16-CD56brightCD14-CD3-CD19-CD20-)
