# Supplementary material for: A hypofractionated radiation regimen avoids the lymphopenia associated with neoadjuvant chemoradiation therapy of borderline resectable and locally advanced pancreatic adenocarcinoma
Source: J Immunother Cancer. 2016 Aug 16;4:45. doi: 10.1186/s40425-016-0149-6 (PMC4986363; doi:10.1186/s40425-016-0149-6)
Supplement: Additional file 3: Table S2. — NCI CTCAE v4.0 hematological toxicity - fractionated. (DOCX 20 kb) [file 40425_2016_149_MOESM3_ESM.docx]

Additional file 3: **Table S2. NCI CTCAE v4.0 hematological toxicity - fractionated**

|  | **Neutrophils** | | | **WBC** | | | **Platelets** | | | **HGB** | | |
| --- | --- | --- | --- | --- | --- | --- | --- | --- | --- | --- | --- | --- |
| **Sample** | **Grade 1-2** | **Grade 3** | **Grade 4** | **Grade 1-2** | **Grade 3** | **Grade 4** | **Grade 1-2** | **Grade 3** | **Grade 4** | **Grade 1-2** | **Grade 3** | **Grade 4** |
| Screening | 0 | 0 | 0 | 0 | 0 | 0 | 2 | 0 | 0 | 5 | 0 | 0 |
| D1 | 0 | 0 | 0 | 0 | 0 | 0 | 3 | 0 | 0 | 5 | 0 | 0 |
| D8 | 3 | 1 | 0 | 5 | 0 | 0 | 4 | 0 | 0 | 8 | 0 | 0 |
| D15 | 5 | 3 | 0 | 5 | 2 | 0 | 7 | 0 | 0 | 7 | 0 | 0 |
| D29 | 0 | 0 | 0 | 0 | 0 | 0 | 2 | 0 | 0 | 8 | 0 | 0 |
| D36 | 0 | 0 | 0 | 2 | 0 | 0 | 2 | 0 | 0 | 8 | 0 | 0 |
| D43 | 5 | 0 | 0 | 6 | 0 | 0 | 4 | 2 | 0 | 8 | 0 | 0 |
| D50 | 2 | 0 | 0 | 5 | 0 | 0 | 6 | 1 | 0 | 8 | 0 | 0 |
| D57 | 4 | 0 | 0 | 6 | 0 | 0 | 3 | 1 | 0 | 9 | 0 | 0 |
| D64 | 2 | 0 | 0 | 6 | 0 | 0 | 1 | 1 | 0 | 8 | 0 | 0 |
| D71 | 1 | 0 | 0 | 4 | 1 | 0 | 3 | 0 | 0 | 7 | 1 | 0 |
| D78 | 2 | 0 | 0 | 3 | 0 | 0 | 3 | 0 | 0 | 7 | 0 | 0 |
| D88 | 0 | 0 | 0 | 2 | 0 | 0 | 2 | 0 | 0 | 6 | 0 | 0 |
| PRD 1 | 2 | 0 | 0 | 3 | 0 | 0 | 2 | 0 | 0 | 7 | 0 | 0 |
| PRD 8 | 2 | 3 | 0 | 1 | 4 | 0 | 4 | 0 | 0 | 6 | 1 | 0 |
| PRD 15 | 0 | 2 | 0 | 3 | 1 | 0 | 2 | 0 | 0 | 7 | 0 | 0 |
| PRD 29 | 0 | 1 | 0 | 0 | 1 | 0 | 3 | 0 | 0 | 6 | 0 | 0 |
| PRD 36 | 0 | 1 | 0 | 3 | 1 | 0 | 3 | 1 | 0 | 4 | 1 | 0 |
| PRD 43 | 2 | 0 | 0 | 3 | 0 | 0 | 1 | 1 | 0 | 5 | 0 | 0 |
| PRD 57 | 0 | 1 | 0 | 1 | 1 | 0 | 2 | 0 | 0 | 6 | 0 | 0 |
| FU 1 | 0 | 1 | 0 | 1 | 1 | 0 | 3 | 0 | 0 | 5 | 0 | 0 |
| FU 2 | 0 | 0 | 0 | 2 | 0 | 0 | 3 | 0 | 0 | 5 | 0 | 0 |
| FU 3 | 0 | 0 | 0 | 1 | 0 | 0 | 2 | 0 | 0 | 4 | 0 | 0 |
| FU 4 | 0 | 0 | 0 | 0 | 0 | 0 | 2 | 0 | 0 | 4 | 0 | 0 |
| FU 5 | 0 | 0 | 0 | 0 | 0 | 0 | 1 | 0 | 0 | 4 | 0 | 0 |
| FU 6 | 1 | 0 | 0 | 0 | 1 | 0 | 2 | 0 | 0 | 3 | 0 | 0 |
