# Supplementary material for: A hypofractionated radiation regimen avoids the lymphopenia associated with neoadjuvant chemoradiation therapy of borderline resectable and locally advanced pancreatic adenocarcinoma
Source: J Immunother Cancer. 2016 Aug 16;4:45. doi: 10.1186/s40425-016-0149-6 (PMC4986363; doi:10.1186/s40425-016-0149-6)
Supplement: Additional file 4: Table S3. — NCI CTCAE v4.0 hematological toxicity - hypofractionated. (DOCX 20 kb) [file 40425_2016_149_MOESM4_ESM.docx]

Additional file 4: **Table S3. NCI CTCAE v4.0 hematological toxicity - hypofractionated**

|  | **Neutrophils** | | | **WBC** | | | **Platelets** | | | **HGB** | | |
| --- | --- | --- | --- | --- | --- | --- | --- | --- | --- | --- | --- | --- |
| **Sample** | **Grade 1-2** | **Grade 3** | **Grade 4** | **Grade 1-2** | **Grade 3** | **Grade 4** | **Grade 1-2** | **Grade 3** | **Grade 4** | **Grade 1-2** | **Grade 3** | **Grade 4** |
| Screening | 0 | 0 | 0 | 2 | 0 | 0 | 1 | 0 | 0 | 7 | 0 | 0 |
| D1 | 0 | 0 | 0 | 0 | 0 | 0 | 1 | 0 | 0 | 7 | 0 | 0 |
| D8 | 6 | 0 | 0 | 6 | 0 | 0 | 3 | 0 | 0 | 10 | 0 | 0 |
| D15 | 3 | 2 | 0 | 5 | 1 | 0 | 5 | 0 | 0 | 9 | 0 | 0 |
| D50 | 0 | 0 | 0 | 2 | 0 | 0 | 4 | 0 | 0 | 9 | 0 | 0 |
| PRD 1 | 0 | 0 | 0 | 1 | 0 | 0 | 0 | 0 | 0 | 5 | 1 | 0 |
| PRD 8 | 4 | 1 | 0 | 6 | 1 | 0 | 0 | 0 | 0 | 8 | 0 | 0 |
| PRD 15 | 3 | 1 | 0 | 5 | 1 | 0 | 3 | 0 | 0 | 8 | 0 | 0 |
| PRD 29 | 0 | 0 | 0 | 0 | 0 | 0 | 0 | 0 | 0 | 7 | 0 | 0 |
| PRD 36 | 3 | 0 | 0 | 1 | 1 | 0 | 0 | 0 | 0 | 7 | 0 | 0 |
| PRD 43 | 1 | 1 | 0 | 1 | 1 | 0 | 1 | 0 | 0 | 6 | 0 | 0 |
| PRD 57 | 0 | 0 | 0 | 0 | 0 | 0 | 0 | 0 | 0 | 5 | 1 | 0 |
| PRD 64 | 2 | 0 | 0 | 1 | 1 | 0 | 0 | 0 | 0 | 5 | 0 | 0 |
| PRD 71 | 3 | 0 | 0 | 3 | 0 | 0 | 3 | 0 | 0 | 5 | 0 | 0 |
| PRD 85 | 0 | 0 | 0 | 0 | 0 | 0 | 1 | 0 | 0 | 5 | 0 | 0 |
| FU 1 | 0 | 0 | 0 | 1 | 0 | 0 | 1 | 0 | 0 | 7 | 0 | 0 |
| FU 2 | 1 | 0 | 0 | 1 | 0 | 0 | 1 | 0 | 0 | 2 | 0 | 0 |
| FU 3 | 1 | 0 | 0 | 1 | 0 | 0 | 1 | 0 | 0 | 1 | 0 | 0 |
| FU 4 | 0 | 0 | 0 | 1 | 0 | 0 | 0 | 0 | 0 | 1 | 0 | 0 |
| FU 5 | 1 | 0 | 0 | 0 | 0 | 0 | 1 | 0 | 0 | 0 | 0 | 0 |
| FU 6 | 1 | 0 | 0 | 0 | 0 | 0 | 1 | 0 | 0 | 1 | 0 | 0 |
